# Establishment of a multimarker qPCR panel for the molecular characterization of circulating tumor cells in blood samples of metastatic breast cancer patients during the course of palliative treatment

**DOI:** 10.18632/oncotarget.9528

**Published:** 2016-05-20

**Authors:** Maren Bredemeier, Philippos Edimiris, Mitra Tewes, Pawel Mach, Bahriye Aktas, Doreen Schellbach, Jenny Wagner, Rainer Kimmig, Sabine Kasimir-Bauer

**Affiliations:** ^1^ Department of Gynecology and Obstetrics, University Hospital Essen, University of Duisburg-Essen, Essen, Germany; ^2^ Department of Medical Oncology, West German Cancer Center, University Hospital Essen, University of Duisburg-Essen, Essen, Germany; ^3^ QIAGEN Hannover GmbH, Langenhagen, Germany

**Keywords:** circulating tumor cells, multimarker gene panel, metastatic breast cancer

## Abstract

**Background:**

Circulating tumor cells (CTC) are discussed to be an ideal surrogate marker for individualized treatment in metastatic breast cancer (MBC) since metastatic tissue is often difficult to obtain for repeated analysis. We established a nine gene qPCR panel to characterize the heterogeneous CTC population in MBC patients including epithelial CTC, their receptors (EPCAM, ERBB2, ERBB3, EGFR) CTC in Epithelial-Mesenchymal-Transition [(EMT); PIK3CA, AKT2), stem cell-like CTC (ALDH1) as well as resistant CTC (ERCC1, AURKA] to identify individual therapeutic targets.

**Results:**

At TP0, at least one marker was detected in 84%, at TP1 in 74% and at TP2 in 79% of the patients, respectively. The expression of ERBB2, ERBB3 and ERCC1 alone or in combination with AURKA was significantly associated with therapy failure. ERBB2 + CTC were only detected in patients not receiving ERBB2 targeted therapies which correlated with no response. Furthermore, patients responding at TP2 had a significantly prolonged overall-survival than patients never responding (*p* = 0.0090).

**Patients and Methods:**

2 × 5 ml blood of 62 MBC patients was collected at the time of disease progression (TP0) and at two clinical staging time points (TP1 and TP2) after 8–12 weeks of chemo-, hormone or antibody therapy for the detection of CTC (AdnaTest EMT-2/StemCell Select™, QIAGEN Hannover GmbH, Germany). After pre-amplification, multiplex qPCR was performed. Establishment was performed using various cancer cell lines. PTPRC (Protein tyrosine phosphatase receptor type C) and GAPDH served as controls.

**Conclusions:**

Monitoring MBC patients using a multimarker qPCR panel for the characterization of CTC might help to treat patients accordingly in the future.

## INTRODUCTION

Circulating tumor cells (CTC) are suggested as potential surrogate markers for minimal residual disease, the precursor of metastatic disease. Their presence and persistence in blood has been associated with worse outcome in early and metastatic breast cancer (MBC) [1–13]. Furthermore, stem cell-like tumor cells as well as CTC undergoing Epithelial-Mesenchymal-Transition (EMT) have been identified within the population of CTC in these patients [11–20]. At present, there is no standard defined for the selection and detection of this highly heterogeneous population. Most methods require an enrichment step prior to detection, including density gradient centrifugation, and/or positive/negative immunomagnetic procedures through antibodies specific for epithelial markers, such as the epithelial cell adhesion molecule (EPCAM) or leucocytes. New selection technologies include microfluidic devices, e.g. isolation of tumor cells by size using filters and the so called “CTC-Chips”, using antibody-coated microspots. Subsequent characterization of CTC can be performed directly by using cytological approaches or indirectly, applying molecular methods and protein assays [21, 22]. Currently, the CellSearch system^®^ (Veridex LLC) based on immunomagnetic EPCAM capturing is the only FDA approved system for CTC enumeration and the most frequently used one in the majority of the above mentioned clinical studies. However, the SWOG-S0500 trial showed no difference in the overall survival (OS)of patients with an increase in CTC counts during the course of therapy when either maintaining or changing therapy [23]. Despite the prognostic impact of CTC counts, characterization of CTC might complement these studies by improving the overall detection rate as well as sensitivity and thus permitting the assessment of potentially predictive molecular markers in CTC of MBC patients as already shown [24].

Molecular characterization of CTC is important not only to confirm their malignant origin but also to follow immuno-phenotypic changes with tumor progression and to identify diagnostically and therapeutically relevant targets which will help to select cancer patients for individual therapies. In this regard, ERBB2 as well as ER/PR were shown to be differentially expressed between the primary tumor and corresponding metastases and/or CTC and the expression status was shown to change during disease progression [25–32]. Furthermore, recently published studies demonstrated mutations in single CTC or bulk of CTC that changed during the course of disease [33–35].

Since metastatic tissue is often difficult to obtain, especially for repeated analysis, a comprehensive analysis of CTC would be appreciable to identify markers which reflect these changes and may allow physicians to tailor treatment accordingly. Most of the already published molecular methods, including ours, have analyzed a subset of genes, e.g. PIK3CA, AKT2, TWIST1, ALDH1, EPCAM, ERBB2, MUC1 and EGFR, respectively [14, 36–40]. Studies using a multimarker qPCR panel to follow up patients during the course of disease have rarely been published up to now [41].

Assuming that the heterogeneous group of CTC consists of epithelial, stem cell-like, EMT-like as well as CTC expressing resistance markers or therapeutic relevant receptors, we validated a qPCR multimarker gene panel [EPCAM, PIK3CA, AKT2, ERCC1, AURKA (Aurora Kinase A), ERBB2, ERBB3, EGFR, ALDH1] for the characterization of CTC in blood samples of MBC patients during palliative treatment. It was the purpose of the study to correlate these findings with response to therapy and to identify targets on CTC that may be able to improve treatment decisions.

## RESULTS

### AdnaPanel Breast expression profile in healthy donors

The raw data (ct-values) of 17 healthy donor (HD) profiles using the AdnaPanel Breast are summarized in Supplementary Figure 1. The ct-values shown for each gene represent the mean of 17 different HD samples. From these data, the cut-off values were calculated as ct mean-(1) 2 × standard deviation and are displayed as grey squares in the graph. All genes were detected at a mean ct-value ranging from 25 to 32, except for the housekeeping gene GAPDH which was detected at lower ct values.

### Specificity determination of the target genes

Applying the cut-off values generated within HD expression profiles (see above) in the calculation algorithm, the specificity was calculated for each gene as (1-N false positive/N all)*100. For ERBB2 and PIK3CA, a specificity of 100% was reached. Except for AURKA and EGFR, all specificities were about 90% and above 80% for AURKA and EGFR (Supplementary Figure 2)

### Influence of contaminating leucocytes

Assuming that several genes of interest are not exclusively expressed in CTC but also to a certain amount in contaminating leucocytes (approximately 150 leucocytes per sample), a PTPRC (Protein tyrosine phosphatase receptor type C, also known as CD45) normalizer was used to calculate the leucocyte contribution of each gene building up a ΔΔCt. Leucocyte titration experiments showed that a growing number of leucocytes is linear to the expression intensity of PTPRC, resulting in a background signal dependent on the leucocyte amount in each sample (data not shown). Thus, the following calculation was performed: ΔΔCt (= (Cut-off_(gene)_-Sample) Ct(_gene)_-(Cut-off_(PTPRC)_-Sample Ct(_(PTPRC)_).

### Gene expression in cancer cell lines

Ten or 20 cells of each cell line were spiked into the blood of HD and treated in the same way as described for patient samples. As shown in Supplementary Figure 3, the cell lines tested reflected the different CTC phenotypes. Among the various cell lines tested, the prostate cancer cell line LNCAP expressed most marker of interest and was used for further assay establishment Supplementary Figure 4. An artificial positive control was used for validation of ERCC1 expression and panel adjustment (Supplementary Figure 5).

### Gene expression in MBC patients

**Figure 1:**
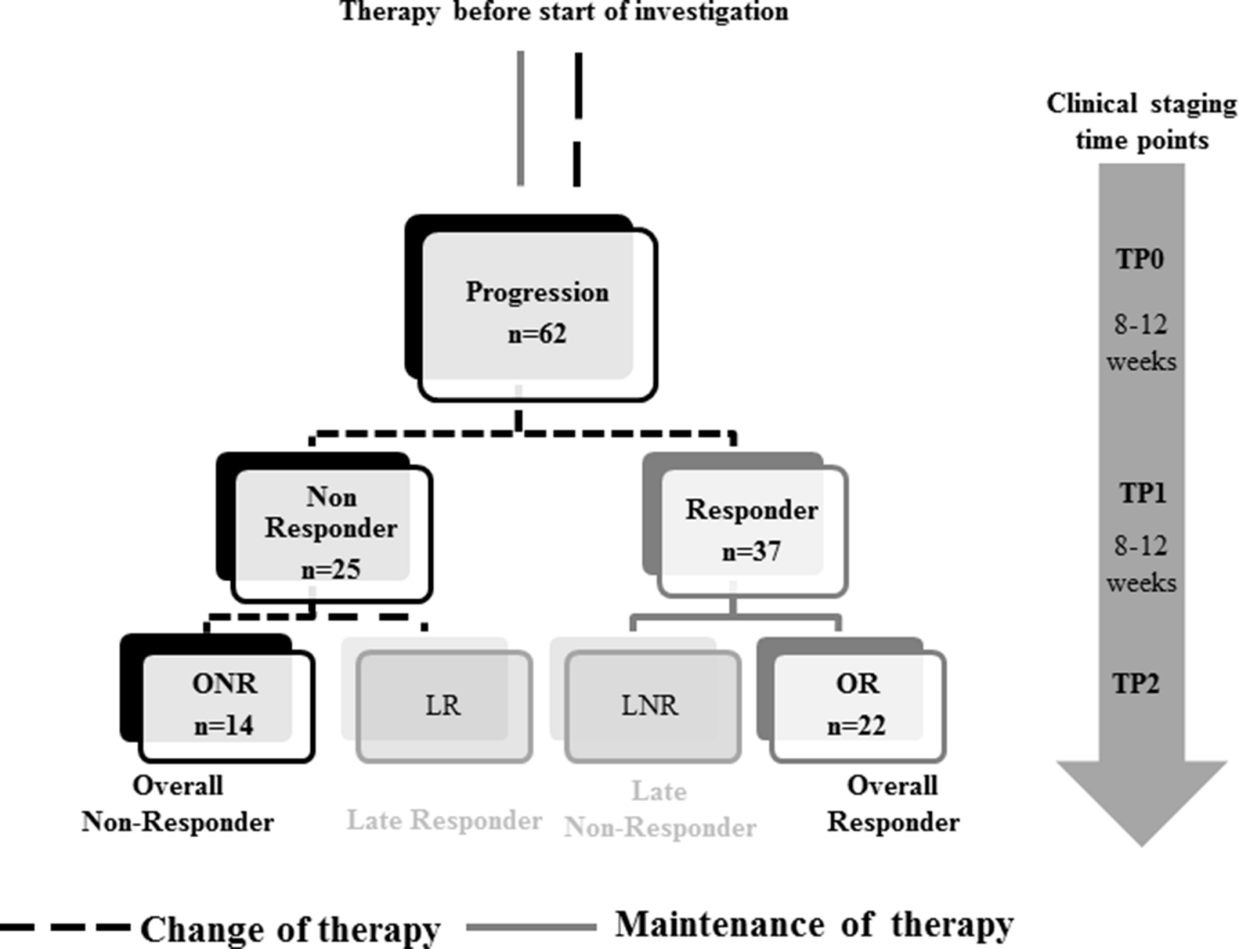
Study design Blood was collected at the time of disease progression (TP0) and at two consecutive clinical staging time points (TP1 and TP2). Patients were stratified according to response to therapy into Responder/Non-Responder at TP1 and in Overall-Responder (OR; Response at TP1 and TP2) and Overall-Non-Responder (ONR; No response at TP1 and TP2), as well as Late Responder (LR; No response at TP1, response at TP2) and Late Non-Responder (LNR; Response at TP1, no response at TP2) at TP2.

**Figure 2:**
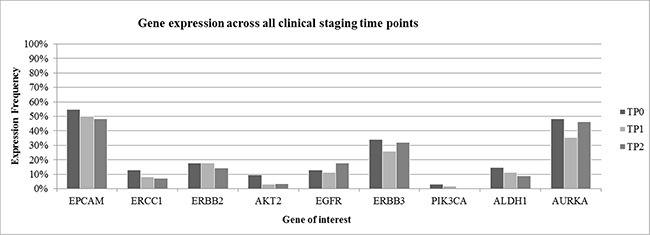
Gene expression across all clinical staging time points Gene expression pattern was highly consistent across all clinical staging time points. EPCAM, AURKA and ERBB3 were the most commonly expressed genes. Clinical outcome was not taken into consideration to get a general overview of gene expression in patient samples.

The study design is shown in Figure 1. In total, evaluation of CTC was feasible in 62 patients at TP0 and TP1 and in 56 patients at TP2, respectively. At TP0, at least one of the studied markers was detected in 52/62 (84%) patients, at TP1 in 46/62 (74%) patients and at TP2 in 44/56 (79%) patients, respectively (data not shown). Interestingly, CTC negative patients at TP0 were mostly showing response to therapy. Among these patients, 17 were Overall Responders (OR), followed by nine Late Non-Responders (LNR) and seven Late Responders (LR) and two Overall Non-Responders (ONR). As apparent from Figure 2, gene expression was highly consistent across all time points. EPCAM was the most commonly expressed gene in 51% of all patient samples, followed by AURKA (43%) and ERBB3 (30%), respectively. ERBB2, EGFR and ALDH1 were expressed in 10%–20% of cases whereas the expression of ERCC1, AKT2 and PIK3CA was below 10%.

### Prognostic relevance of the different genes

Table 1 summarizes the prognostic significance of single genes as well as combinations at TP1 and TP2 with regard to response to therapy. In general, the expression of ERBB2 and ERBB3 alone or in combination with AURKA or EGFR was associated with therapy failure. In addition, ERCC1 alone or in combination with ERBB2 or AURKA was of prognostic significance as well as the combination of AURKA and EGFR (*p*-values < 0.05). All other genes, alone or in combination, showed no prognostic significance with regard to response at TP1 or TP2.

**Table 1:** Prognostic relevance of the different genes analyzed

	After therapy (TP1)	After therapy (TP2)
Genes detected in CTC	*p-*value	*p-*value
EPCAM	0,1955	**0,0330**
ERCC1	**0,0082**	0,2787
ERBB2	**0,0029**	**0,0196**
ERBB3	**0,0357**	**0,0133**
**Combined gene expression**		
ERBB3 + AURKA	0,0814	**0,0145**
ERBB2 + AURKA	**0,0404**	**0,0337**
ERBB2 + ERCC1	**0,0226**	0,1055
ERBB2 + EGFR	0,1586	**0,0477**
AURKA + ERCC1	**0,0226**	0,2787
AURKA + EGFR	0,9877	**0,0408**

The expression of some genes and gene combinations was significantly prognostic for having not responded to therapy at TP1 or TP2. Only significant *p*-values of genes/combinations are listed.

### Gene expression according to response groups

**Figure 3:**
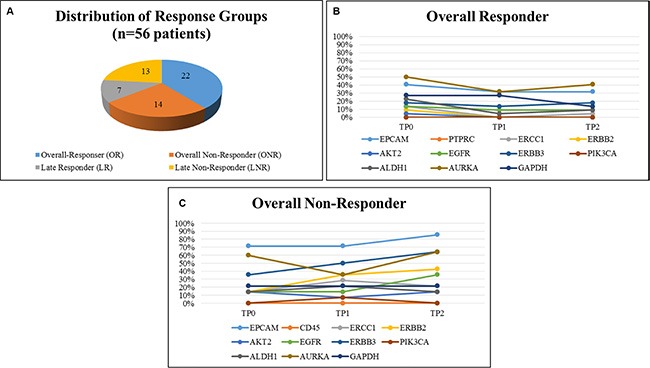
Distribution of response groups and comparison of gene expression in OR and ONR 56 Patients could be evaluated for this analysis and were stratified into 22 OR, 14 ONR, 7 LR and 13 LNR, respectively (**A**). OR showed a consistent low expression of our marker genes, except for AURKA and EPCAM (**B**), in comparison to ONR showing higher gene expression levels at all time points (**C**).

**Figure 4:**
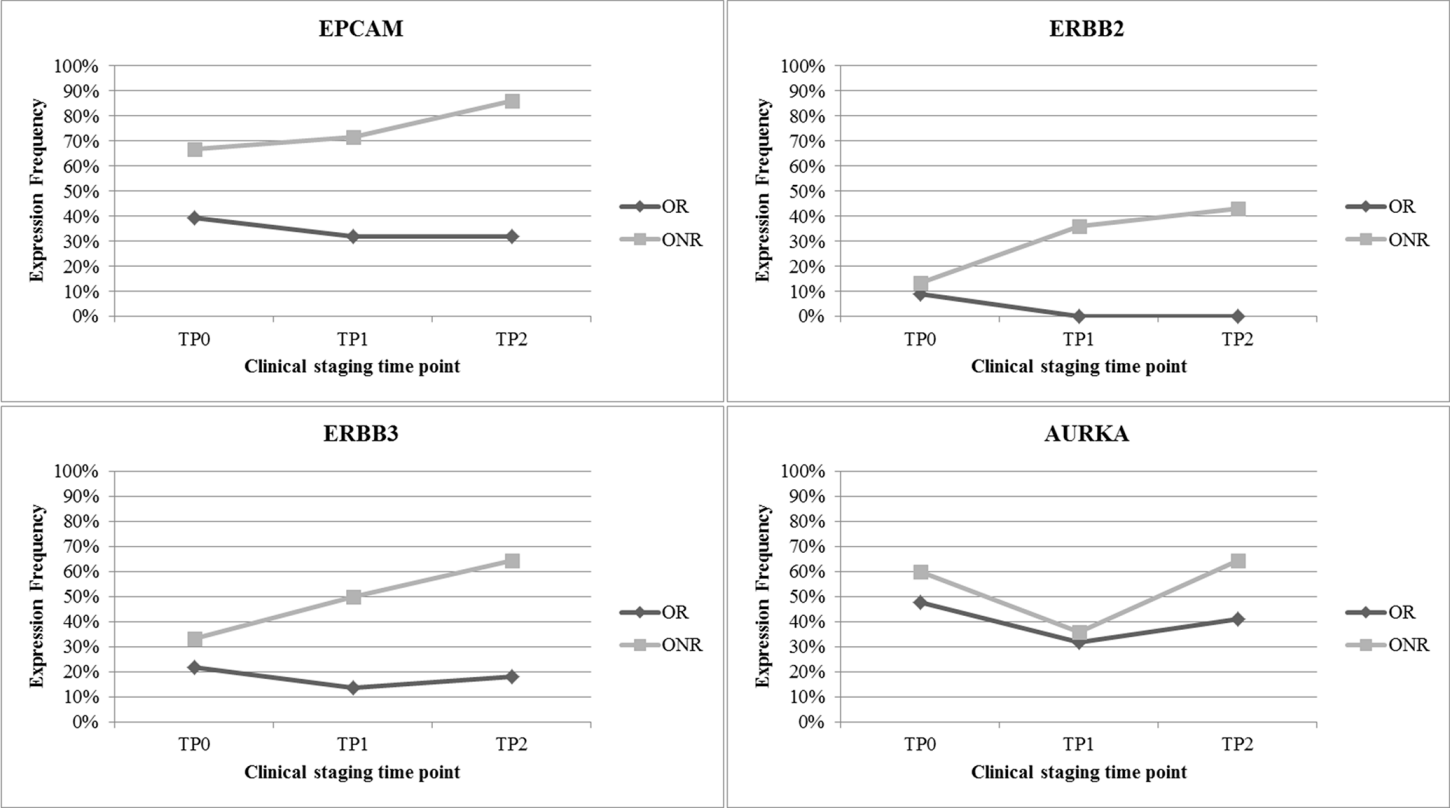
Most differently expressed genes in OR versus ONR EPCAM was expressed in up to 90% of all ONR. Furthermore, great differences in gene expression in OR versus ONR were observed for ERBB2, ERBB3 and AURKA.

As demonstrated in Figure 3A, 22 patients were classified as OR, 14 patients as ONR, seven patients as LR and 13 patients as LNR, respectively. When OR were compared with ONR, OR showed a consistent lower expression of the majority of marker genes across all time points (expression of 0%–30%) except for EPCAM, AURKA, GAPDH… (expression in 30%–50%) (Figure 3B). In contrast, ONR showed enhanced gene expression already at TP0, predominately for EPCAM (70%), AURKA (60%) and ERBB3 (30%), respectively. A continuous increase from TP0 to TP2 was observed for the expression of EPCAM, ERBB2 and ERBB3. Nearly 90% of ONR showed expression of EPCAM at TP2 and expression was significantly associated with not having responded to therapy (*p* = 0.0330) (Figure 3C). The greatest difference in gene expression between the OR and ONR group was observed for EPCAM, ERBB2, ERBB3 and AURKA. Especially for the ONR, a steady increase of EPCAM as well as ERBB2 and ERBB3 expression was observed in comparison to the OR group (Figure 4).

### Correlation of gene expression with outcome

**Figure 5:**
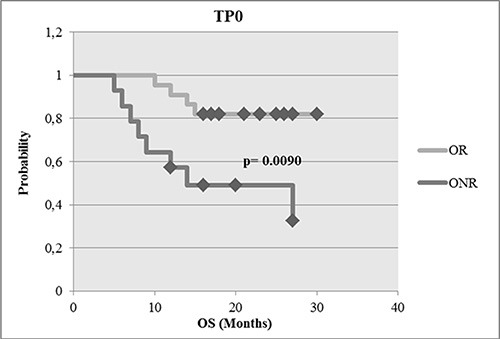
Survival analysis of OR compared with ONR OR had a significant longer OS than ONR at the time point tested (*p* = 0.0090).

The OS was calculated as the period of time from the date of sample drawing (TP0) until the date of death. For OS analysis, only OR and ONR were compared. The median OS was 27 months for OR [*n* = 22, 10 to 30 months] vs. 18 months for ONR [*n* = 14, 5 to 27 months]. As shown in Figure 5, OR had a significantly longer OS than ONR (*p* = 0.0090). As apparent from Table 2, the negative prognostic effect at TP1 seemed to be mostly related to the expression of ERCC1 (*p* = 0.0031) alone or in combination with ERBB2 (*p* = 0.0293) or ERBB3 (*p* = 0.0084) or AURKA (*p* = 0.0094) as well as to the expression of EGFR alone (*p* = 0.0084), or in combination with ERBB3 (*p* = 0.0084) or AURKA (*p* = 0.0084). For responders, no significant single genes or combinations could be identified.

**Table 2:** Genes associated with reduced OS at TP1

Gene expression in Non-Responders at TP1	*p*-value	Hazard ratio	95% CI of ratio
ERCC1	0,0031	0.04723	0.006212 to 0.3591
EGFR	0,0084	0.01274	0.0004959 to 0.3274
ERCC1 + ERBB2	0,0293	0.4091	0.1590 to 0.6592
ERCC1 + ERBB3	0,0084	0.2727	0.06499 to 0.4805
ERCC1 + AURKA	0,0094	0.3636	0.1135 to 0.6137
EGFR + AURKA	0,0084	0.2727	0.06499 to 0.04805
EGFR + ERBB3	0,0084	0.2727	0.06499 to 0.04805

The genes and gene combinations listed were significantly associated with a shorter OS. For Responders, no significant single gene or combinations could be identified.

### Influence of targeted therapies on CTC

**Figure 6:**
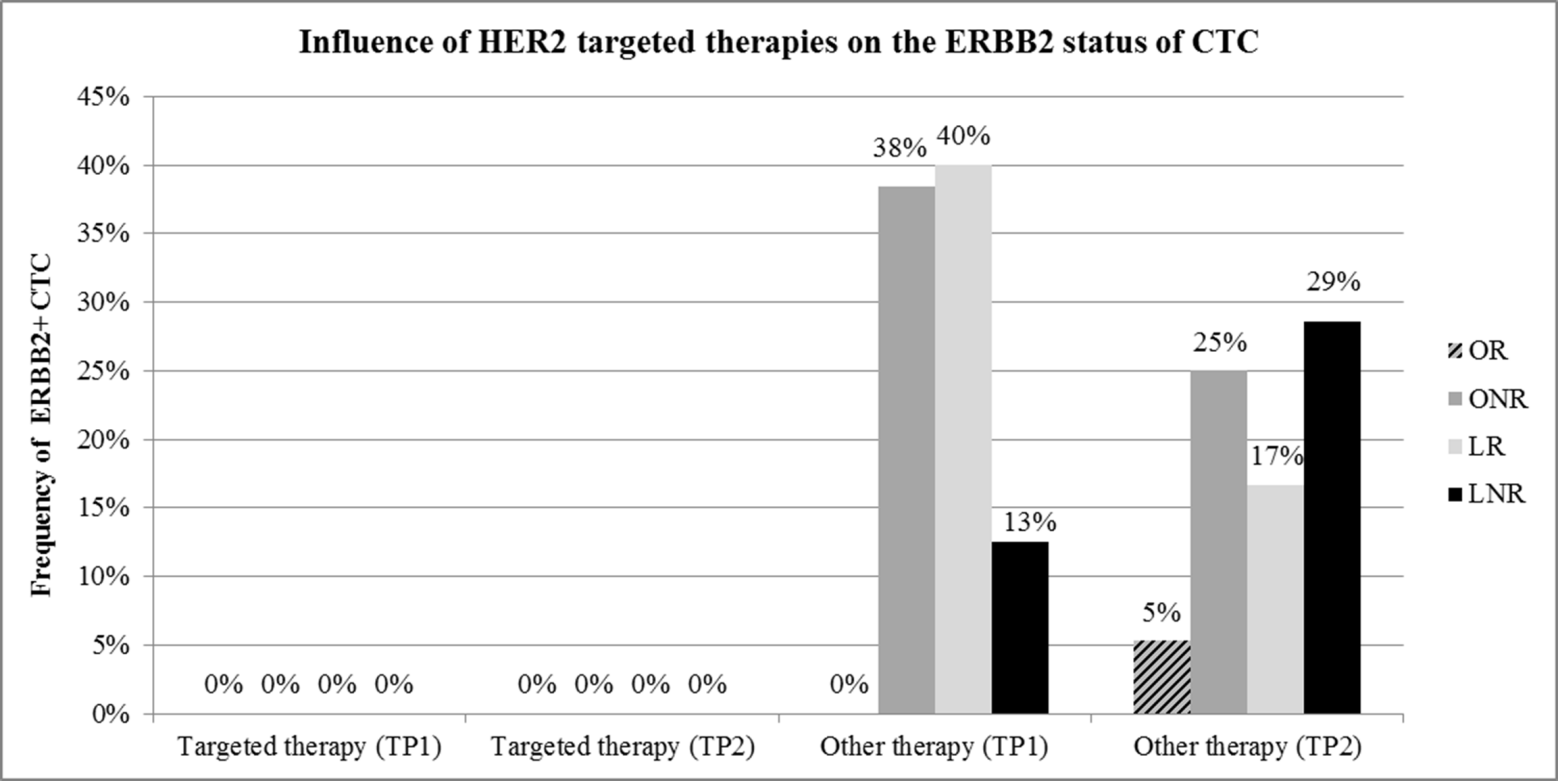
Influence of ERBB2 targeted therapies on the ERBB2 status of CTC No ERBB2 positive CTC (ERBB2+) were detected in patients under ERBB2 targeted treatment at TP1 and TP2. This finding was independent of the response class. ERBB2+ CTC were frequently detected in patients receiving other than targeted therapies, mainly in patients not responding to treatment.

ERBB2 was one of the genes mostly associated with worse outcome. Since 17 patients received ERBB2 targeted therapies during the course of the disease, we evaluated ERBB2 expression in CTC with regard to response to ERBB2 targeted therapy. As apparent from Figure 6, in patients under ERBB2 targeted therapy, no ERBB2 positive CTC were detected, irrespective of the response class. In contrast, in patients not receiving ERBB2 targeted therapy, ERBB2 positive CTC were frequently detected in all response groups except for most of the OR.

## DISCUSSION

### Key findings

In this study, we have established a multimarker qPCR panel to characterize the heterogeneous CTC population to monitor palliative treatment of MBC patients. The most commonly expressed gene was EPCAM, followed by AURKA. Mainly ERBB2/ERBB3 positive CTC as well as CTC expressing the resistance marker AURKA and ERCC1 were associated with worse outcome. In addition, ERBB2 positive CTC were only expressed in patients not receiving ERBB2 targeted therapy.

### Gene expression

Up to now, a variety of groups have been characterizing CTC on the molecular as well as on the cellular level, mostly the expression of single marker genes, only a few studies have been investigating multi marker gene panel. The comparison of ERBB2 expression on CTC and tumor tissue resulted in an overall concordance of 74% and 89% when comparing CTC with the primary tumor and 69% when compared to metastases, respectively [29, 45].

Assessing six genes in 64 operable BC and 20 MBC patients as well as in 17 HD, Markou et al. detected CK19, ERBB2, MAGEA3, SCGB2A2 and TWISTP1 in 26.6%, 12.5%, 18.7%, 10.9% and 31.2% cases, respectively [46]. An EMT-like as well as stem cell-like CTC phenotype has also been described in other studies. In this regard, using qPCR for the expression of EMT transcription factors, Mego et al., showed that patients with a high percentage of CD326+ or EMT transcription factor overexpressing cells had a shorter progression-free survival [47]. In addition, the EMT transcription factors TWISTP1, SNAIL1, ZEB1, TG2 and the stemness marker ALDH1, CD24, CD44 and CD133 were shown to be present in the CD326−PTPRC− fraction of ERBB2+ MBC patients [10]. Comparable data were also published by others applying confocal laser scanning microscopy for the detection of phosphorylated (phospho) PIK3CA, phospho-EGFR, phospho-AKT and/or ERBB2 in early and MBC patients [36, 48]. “With regard to PIK3CA, hotspot mutations…” were frequently detected in CTC of operable BC and MBC patients which changed during tumor progression resulting in a worse outcome [35]. Using singleplex and multiplex qPCR, our group has already shown that the expression of at least one EMT marker (PIK3CA, AKT2, TWIST1) and/or the expression of ALDH1 might serve as an indicator for therapy resistance resulting in a poor prognosis in MBC patients [14]. However, analyzing CTC more comprehensively, it seems as if the expression of epithelial CTC, resistant CTC, as well as ERBB2 and/or ERBB3 and/or EGFR positive cells seem to be more associated with worse outcome, especially in ONR. This might have different reasons. On the one hand, these findings are in accordance with other studies, showing that feedback mechanisms caused by intervention in the PIK3CA/AKT2 pathway can lead to rebounded ERBB3 activity and hence, therapy resistance [49–52]. On the other hand, the individual therapies might have eradicated stem cell-like CTC as already shown for trastuzumab, lapatinib and everolimus [53–55]. Furthermore, CTC that have undergone EMT might have performed a switch back to epithelial CTC.

A variety of studies have already shown the prognostic impact of CTC as well as their function of monitoring disease progression [1–13, 56, 57]. However, the SWOG-S0500 trial, changing therapy of MBC patients versus maintaining the therapy with regard to changes in CTC counts during follow-up assessment, showed no difference in OS of patients with an increase in CTC counts during course of therapy when either maintaining or changing therapy [23]. Thus, new strategies to target CTC are investigated in clinical studies. The DETECT III study was directed at initial ERBB2- MBC patients with ERBB2+ CTC, investigating the effect of ERBB2 targeted therapy with lapatinib plus standard treatment (ClinicalTrials.gov Identifier: NCT01619111). The succession study DETECT IV was designed for patients with hormone receptor positive but ERBB2- MBC, having ERBB2– CTC. This study is investigating the effect of everolimus in combination with standard treatment on CTC (ClinicalTrials.gov Identifier: NCT02035813).

Despite the prognostic impact of CTC counts, comprehensive molecular profiling might complement these studies by improving the specificity and, thus, permitting the assessment of genomic markers in CTC of MBC patients. In this regard, Mostert et al. developed a 16 gene profile assay based on qPCR mRNA expression quantification, distinguishing patients showing no response to therapy or dying in less than nine months after start of first-line systemic therapy from those with a better prognosis [58]. Moreover, Fina et al. compared CTC counts with gene expression patterns based on the PAM50 gene panel in CTC from seven MBC patients resulting in no correlation between CTC number and gene expression levels as well as molecular subtype specific differences [41].

Monitoring CTC over a period of time seems necessary since marker profiles can change during course of a therapy and during disease progression as already shown for ERBB2, ER and PR [27, 32, 59]. For ERBB2, several studies indicated that ERBB2+ MBC patients have EMT-like CTC and that ERBB2 seems to be selectively expressed in cancer stem cells of initially ER+ and ERBB2-negative luminal BC [10, 55]. There is growing evidence that patients with an ERBB2-negative primary tumor could also benefit from a trastuzumab treatment if ERBB2+ CTC can be detected [30, 60–61]. This is in accordance with our study showing that ERBB2+ cells were only found in patients not receiving ERBB2 targeted treatment whereas patients under an ERBB2 targeted therapy had no ERBB2+ CTC. Very recently, Kallergi et al. showed that the expression of truncated ERBB2 (p95ERBB2) on CTC of BC patients, was associated with a poor prognosis and resistance to trastuzumab therapy [62].

Moreover, the expression of ERBB2 and ERBB3 alone or in combination with resistance markers seem to promote uncontrolled tumor cell growth in overt metastases, leading to a shorter OS. Several studies are currently investigating the effect of combining ERBB2 and/or ERBB3 treatment with conventional therapy. As an example, the PERUSE study is evaluating the treatment of MBC or locally advanced BC patients with pertuzumab in combination with trastuzumab and taxane in first-line (ClinicalTrials.gov Identifier: NCT01572038). Similarly the CLEOPATRA study is investigating the improvement in the OS of ERBB2+ MBC patients when treated with a combination of pertuzumab, trastuzumab and chemotherapy with docetaxel (ClinicalTrials.gov Identifier NCT00567190).

We were also able to show that EGFR was frequently expressed in CTC of non-responders which was associated with a shorter OS whereas the expression of AURKA was associated with a shorter OS when comparing OR with ONR. With regard to AURKA, these results are in alignment with the results of Zhang et al. who demonstrated that AURKA expression was significantly associated with a shorter OS by systematic review and meta-analysis in various solid tumors. For BC exclusively, the AURKA expression levels were not significantly associated with poor prognosis but patients receiving the AURKA inhibitor alisertib in a phase II study, showed significant response [63].

EGFR and downstream pathways were shown to be implicated in the regulation of EMT and invasion and EGFR overexpression has been observed across all BC subtypes [64–70]. Tyrosinkinase inhibitors were shown to inhibit invasion and cell motility by transforming mesenchymal cells to an epithelial phenotype and thus might be acting through inhibition of EMT *in vitro* [71]. However, results of clinical studies have often been disappointing probably due to the fact that patients were not selected on the basis of EGFR expression and had a long therapy history [72, 73].

## MATERIALS AND METHODS

### Patient population and characteristics

The study was conducted at the Department of Gynecology and Obstetrics in collaboration with the Department of Internal Medicine (Cancer Research) at the University Hospital Essen. In total, 180 samples of 62 MBC patients have been studied from November 2013 until May 2015.

### Eligibility criteria

The eligibility criteria were as follows: age ≥ 18 years; measurable or evaluable MBC; predicted life expectancy ≥ 2 months; Eastern Cooperative Oncology Group (ECOG) scores for performance status of 0–2; no severe uncontrolled co-morbidities or medical conditions; no second malignancies. Patients had either a relapse of breast cancer diagnosed years before and were to start chemotherapy or a documented progressive BC before receiving a new endocrine, chemo- or experimental therapy. Prior adjuvant treatment, radiation or any other treatment of metastatic disease were permitted. Exclusion criteria were other malignancies except breast cancer. All specimens were obtained after written informed consent and collected using protocols approved by the institutional review board (05/2856).

### Response criteria

Before starting a new treatment, patients underwent an evaluation of metastatic sites by ultrasound, x-ray or computer tomography. Re-evaluations of disease status were done by the same techniques every 8–12 weeks, depending on the treatment schedule, until the loss or death of a patient. Response to therapy was evaluated according to the Response Evaluation Criteria in Solid Tumors (RECIST). Complete Response (CR): disappearance of all target lesions; Partial Response (PR): at least 30% decrease in the sum of the LD (longest diameter) of target lesions, taking as reference the baseline sum LD; Progressive Disease (PD): at least 20% increase in the sum of the LD target lesions, taking as reference the smallest sum LD recorded since the treatment started or the appearance of one or more new lesions; Stable Disease (SD): neither sufficient shrinkage to qualify for PR nor sufficient increase to qualify for PD, taking as reference the smallest sum LD since the treatment started.

### Study design

2 × 5 ml blood of 62 MBC patients was collected at three different time points for the evaluation of CTC (Figure 1). TP0: new metastasis of the disease or progressive disease under a given therapy; TP1: 8–12 weeks after chemo-, hormone or antibody therapy; TP2: after further 8–12 weeks of therapy.

### Stratification of patients

Patients were stratified as follows: (1) Overall Responder (OR, response to therapy at TP1 and TP2), (2) Overall Non-Responder (ONR, no response to therapy at TP1 and TP2), (3) Late Responder (LR, response to therapy at TP2 but not at TP1) and (4) Late Non-Responder (LNR, response to therapy at TP1 but not at TP2), respectively. According to RECIST criteria, patients with SD, CR or PR were classified as Responders to therapy whereas patients with PD were Non-Responders.

### Immunohistochemical analysis

For each of the 62 patients, the tumor type, TNM-staging and grading were assessed according to the WHO-classification of tumors of the breast [74] and the sixth edition of the TNM Classification System [75]. The estrogen (ER) and the progesterone (PR) receptor status were determined by immunohistochemistry. The DAKO-score for the expression of ERBB2 was re-evaluated with the HercepTest^®^ (Dako). FISH analysis in cases of 2+ staining as determined with the HercepTest^®^ was performed as described elsewhere [76].

### Assay establishment in cell culture experiments

The multimarker gene panel for CTC characterization was established using cell lines of different tumor entities since not all genes of interest were expressed uniquely in one cell line. 20 cells of LNCAP (prostate), PC3 (prostate), HTP1197 (bladder), RT4 (urinary bladder) and Ovcar3 (ovarian) as well as 10 cells of the breast cancer cell lines MDAMB231, T47D, SKBR3 and MCF- 7 were spiked into blood of healthy donors (HD, *n* = 17) and processed as described below. Among the various cell lines tested, the prostate cell line LNCAP expressed most marker of interest and was used for further assay validation. Since ERCC1 was not expressed by the cell lines tested, an artificial positive control was used for establishment (Supplementary Figures 3, 4 and 5). The cell lines were purchased from the ATCC (American Tissue Culture Collection, Rockville, MD) and cultured in a humidified incubator at 37°C in an atmosphere of 5% CO_2_ and 95% air. The cell lines were maintained in RPMI medium supplemented with 10% heat-inactivated fetal bovine serum and 1% penicillin/streptomycin (Biochrom KG, Seromed, Berlin, Germany). Amplicon sizes and references of the target genes are shown in Supplementary Table 1.

### Sampling of blood

At each time point, 2 × 5 ml EDTA blood was collected for CTC isolation. All “in-house samples” were collected in S-Monovettes^®^ (Sarstedt AG & Co., Nümbrecht, Germany) whereas “overnight samples” were collected in AdnaCollect tubes (QIAGEN Hannover GmbH, Langenhagen, Germany). As proven by the manufacturer, this did not affect the final results. The samples were stored at 4°C until further examination and were processed immediately or not later than four hours after blood withdrawal (24 hours for overnight samples).

### Enrichment of CTC

Blood samples were analyzed for CTC using the AdnaTest EMT-2/StemCellSelect™ (QIAGEN Hannover GmbH, Langenhagen, Germany) that enriches CTC via antibody coated magnetic beads, targeting EPCAM, EGFR and ERBB2. The AdnaTest EMT-2/StemCell antibody setup has proven better sensitivity over the AdnaTest BreastCancer setup (data not shown). Labelled CTC were extracted using a magnetic particle concentrator according to the manufacturer`s instructions (130122 EN) and were subsequently lysed. The cell lysate was stored for a maximum of two weeks at −80°C until further processing.

### Detection of CTC

mRNA was isolated from the cell lysate of pre-enriched tumor cells using the AdnaTest EMT-2/StemCellDetect™ (QIAGEN Hannover GmbH, Langenhagen, Germany) which is based on oligo(dt)25 coated magnetic beads. Reverse transcription was performed using the Sensiscript Reverse Transcription Kit™ (QIAGEN, Hilden, Germany). cDNA was gene specifically pre-amplified using the TATAA Multiplex Grand Master Mix according to in-house designed assays. PCR activation was performed at 95°C for 3 minutes and followed by 16 cycles of denaturation at 95°C for 20 seconds, extension for 20 seconds at 60°C and elongation at 72°C for 3 minutes, respectively. Multiplex qPCR was performed for the following nine markers: EPCAM (epithelial); PIK3CA, AKT2 (EMT); ALDH1 (stem cell); ERCC1, AURKA (resistance markers); ERBB2, ERBB3, EGFR (receptors); PTPRC (Protein tyrosine phosphatase receptor type C, leucocyte control) and GAPDH (housekeeping gene) as well as the synthetic EPCAM fragment as an internal reference using iTaq Universal Supermix SYBR Green Mix™ (Biorad, Hercules, CA, USA). qPCR was performed using the StepOnePlus™ (Life Technologies, Carlsbad, CA, USA) real time system. After PCR activation at 95°C for 3 minutes, the thermal profile of 35 cycles in total was as follows: 20 seconds at 95°C, 20 seconds at 60°C, 30 seconds at 72°C and 1 minute at 95°C. Additionally, melting curves were performed. Remark: The establishment process and the measurement of HD samples were performed with 40 cycles of qPCR. Since 35 cycles were sufficient after cut-off determination, all patient samples were measured with a shorter cycle setup.

### Data evaluation and statistical analysis

Assuming that several genes of interest are not exclusively expressed in CTC, but also in contaminating leucocytes, a PTPRC normalizer was included to calculate the leucocyte contribution for each gene. In leucocyte titration experiments, we found a linear correlation of the leucocyte number and the PTPRC Ct-value as well as a linear contribution to each genes Ct-value depending on the leucocyte number (data not shown). A cut-off value was calculated for each gene separately in a way that the false positive rate in all HD (*n* = 17) was lower than 10% (specificity > 90%, except for AURKA: specificity > 80%). If there were additional melting peaks or melting peaks at the wrong temperature, the Ct-values were excluded. Furthermore, results were not normalized using GAPDH cq values but were corrected for PTPRC. GAPDH expression is resulting from the number of contaminating leucocytes remaining after CTC enrichment. Subsequently, delta delta Ct was calculated as ΔΔCt = (Cut-off_(gene)_-Sample Ct_(gene)_)-(Cut-off_(PTPRC)_-Sample Ct_(PTPRC)_).

## CONCLUSIONS

Using a multimarker qPCR panel for comprehensive molecular characterization of CTC, we here demonstrate that changes in the molecular profile of CTC in the course of therapeutic interventions can determine response to the applied therapy. Since metastatic tissue is often difficult to obtain, CTC may have the potential to serve as liquid biopsy for better therapy guidance. Further investigations will include more genes to get deeper insights into the heterogeneity of CTC during the course of disease.

## SUPPLEMENTARY FIGURES AND TABLES


