# Supplementary material for: Establishment of a multimarker qPCR panel for the molecular characterization of circulating tumor cells in blood samples of metastatic breast cancer patients during the course of palliative treatment
Source: Oncotarget. 2016 May 20;7(27):41677–90. doi: 10.18632/oncotarget.9528 (PMC5173087; doi:10.18632/oncotarget.9528)
Supplement: Supplementary file 1 [file oncotarget-07-41677-s001.pdf]

Establishment of a multimarker qPCR panel for the molecular characterization of circulating tumor cells in blood samples of metastatic breast cancer patients during the course of palliative treatment

Supplementary Materials

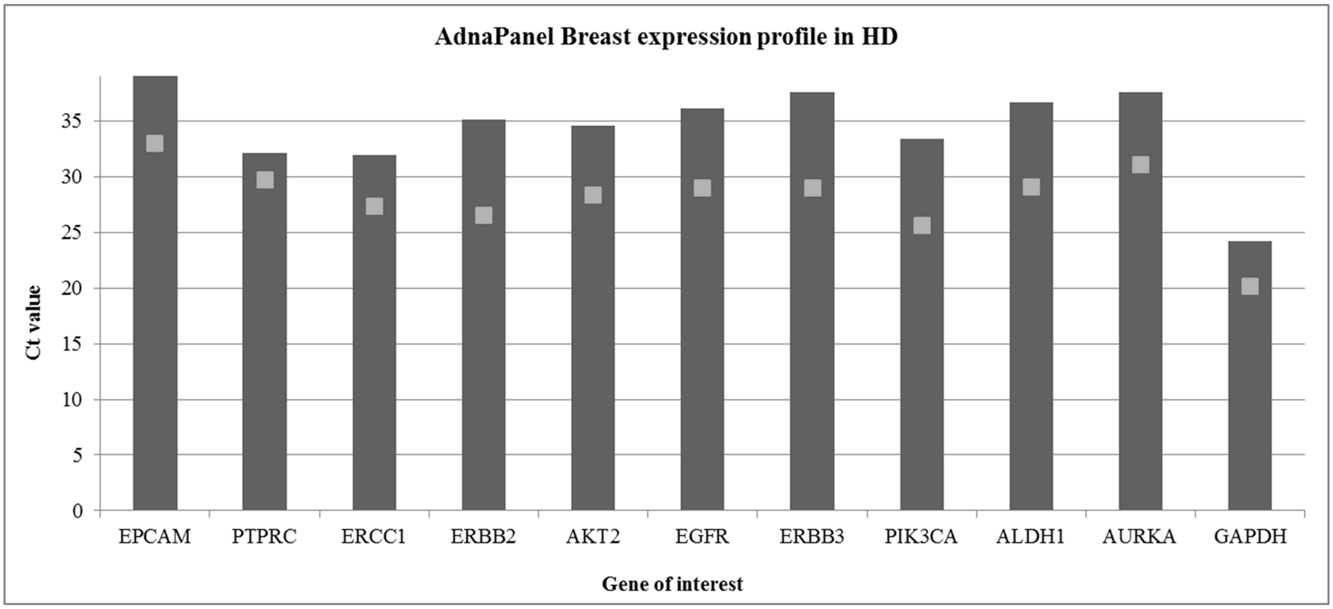

Supplementary Figure S1: AdnaPanel Breast expression profile in HD. 17 HD were tested for a gene specific cut-off determination. The resulting cutt-off values are shown of 17 samples is shown as grey squares. Remark: The establishment process and the measurement of HD samples were performed with 40 cycles of qPCR. Since 35 cycles were sufficient, all patient samples were measured with a shorter cycle setup.

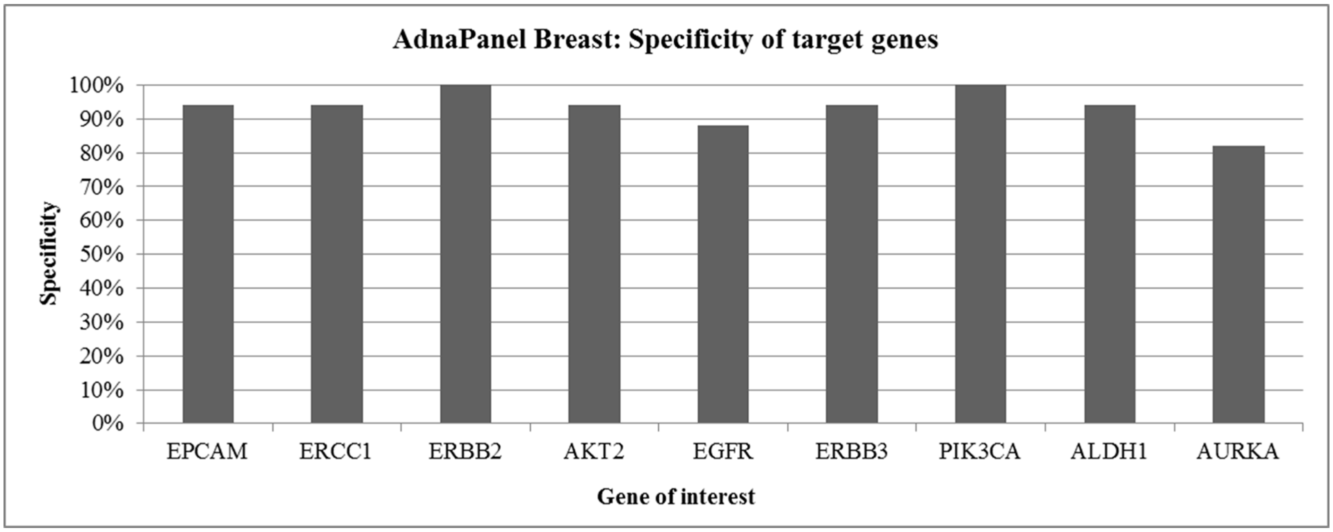

Supplementary Figure S2: AdnaPanel Breast specificity of target genes. Applying the cut-off values determined by testing blood of HD, the specificity for each target gene was calculated.

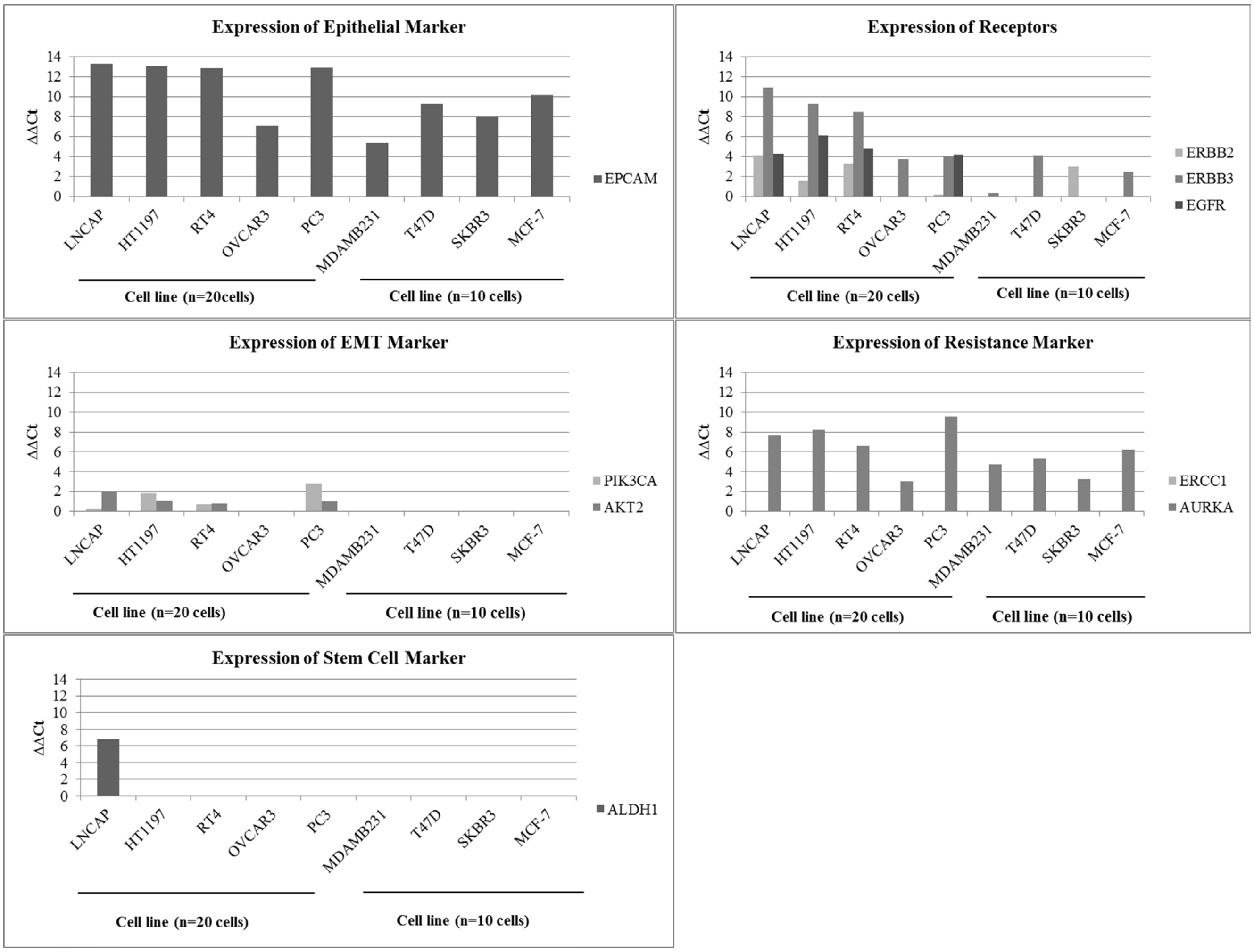

Supplementary Figure S3: Gene expression in cancer cell lines. 10 (MDAMB231, T47D, SKBR3, MCF-7) or 20 cells (LNCAP, HT1197, RT4, OVCAR3, PC3) of each cell line were spiked into 5 ml blood of HD and processed in the same way as patient samples. The cell lines used reflected the different CTC phenotypes.

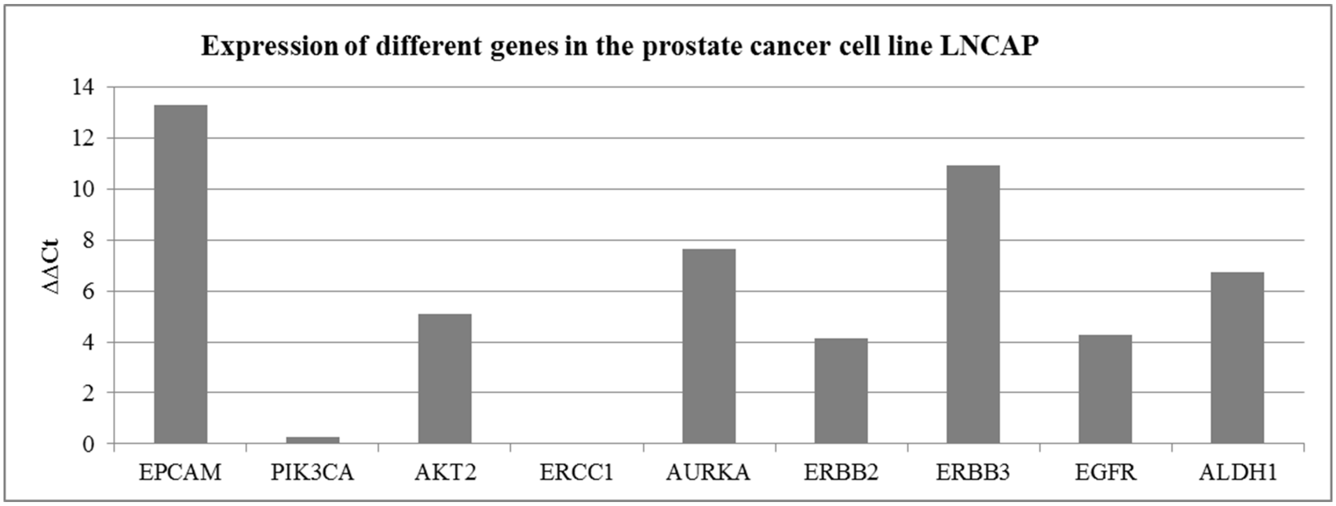

Supplementary Figure S4: Dilution of an artificial positive control. For panel adjustment, especially for the establishment of ERCC1 (not expressed by the cell lines tested), an artificial control was used. 0.07 ng/µl dsDNA were diluted 1:10000. Starting from this basic dilution (1:1), 1:10, 1:100 and 1:1000 dilutions were prepared.

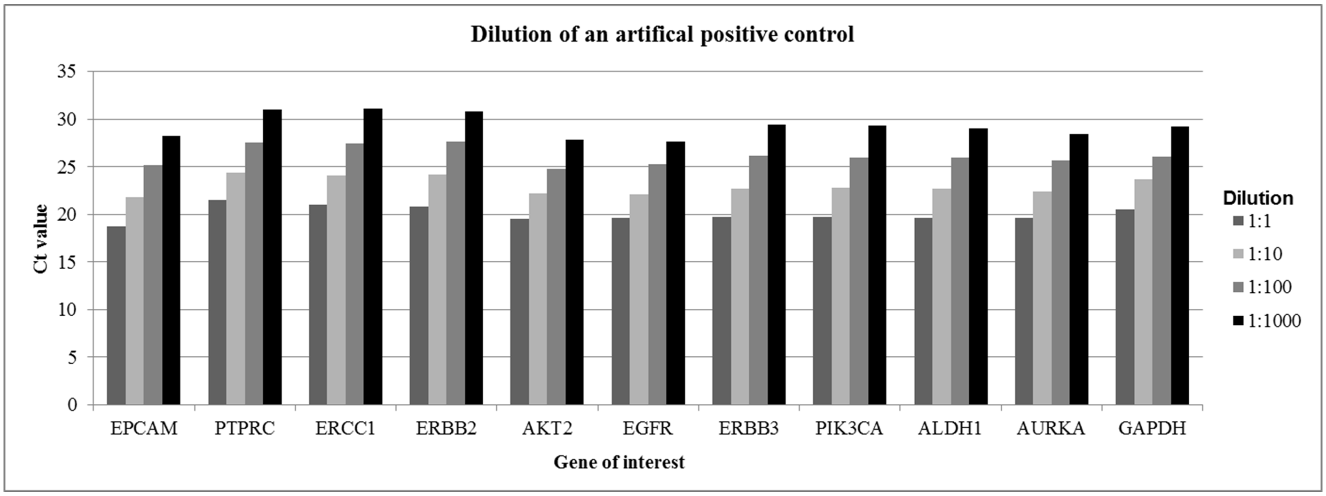

Supplementary Figure S5: Expression of different genes in the prostate cancer cell line LNCAP. For multi marker gene panel establishment, 20 LNCAP cells were spiked into blood of HD. Remark: ERCC1 expression was established using the ERCC1 expresion was established using an artificial positive control.

Supplementary Table S1: Amplicon sizes

Gene	NCBI Ref	Variant	Amplicon size
**EPCAM**	NM_002354.2		156 bp
**ERCC1**	NM_202001.2	Var 1	162 bp
	NM_001983.3	Var 2	
	NM_001166049.1	Var 3	
**PTPRC**	NM_002838.4	Var 1	117 bp
**EGFR**	NM_201282.1	Var 2	137 bp
	NM_005228.3	Var 1	
	NM_201283.1	Var 3	
	NM_201284.1	Var 4	
**AKT2**	NM_001626.4	Var 1	134 bp
	NM_001243027.1	Var 2	
	NM_001243028.1	Var 3	
**ERBB3**	NM_001005915.1	Var s	138 bp
	NM_001982.3	Var 1	
**ALDH1**	NM_000689.4		137 bp
**AURKA**	NM_003600.2	Var 2	157 bp
	NM_198473.1	Var 6	
	NM_198436.1	Var 5	
**PIK3CA**	NM_006218.2		177 bp
**ERBB2**	NM_001005862.1	Var 2	159 bp
	NM_004448.2	Var 1	
**GAPDH**	NM_001001303		75 bp

Supplementary Table S2: Raw qPCR values of patient and of HD samples. See Supplementary_Table_S2
